# Supplementary material for: Dietary Energy Levels Affect Rumen Bacterial Populations that Influence the Intramuscular Fat Fatty Acids of Fattening Yaks (Bos grunniens)
Source: Animals (Basel). 2020 Aug 22;10(9):1474. doi: 10.3390/ani10091474 (PMC7552236; doi:10.3390/ani10091474)
Supplement: Supplementary file 1 [file animals-10-01474-s001.pdf]

Supplementary materials

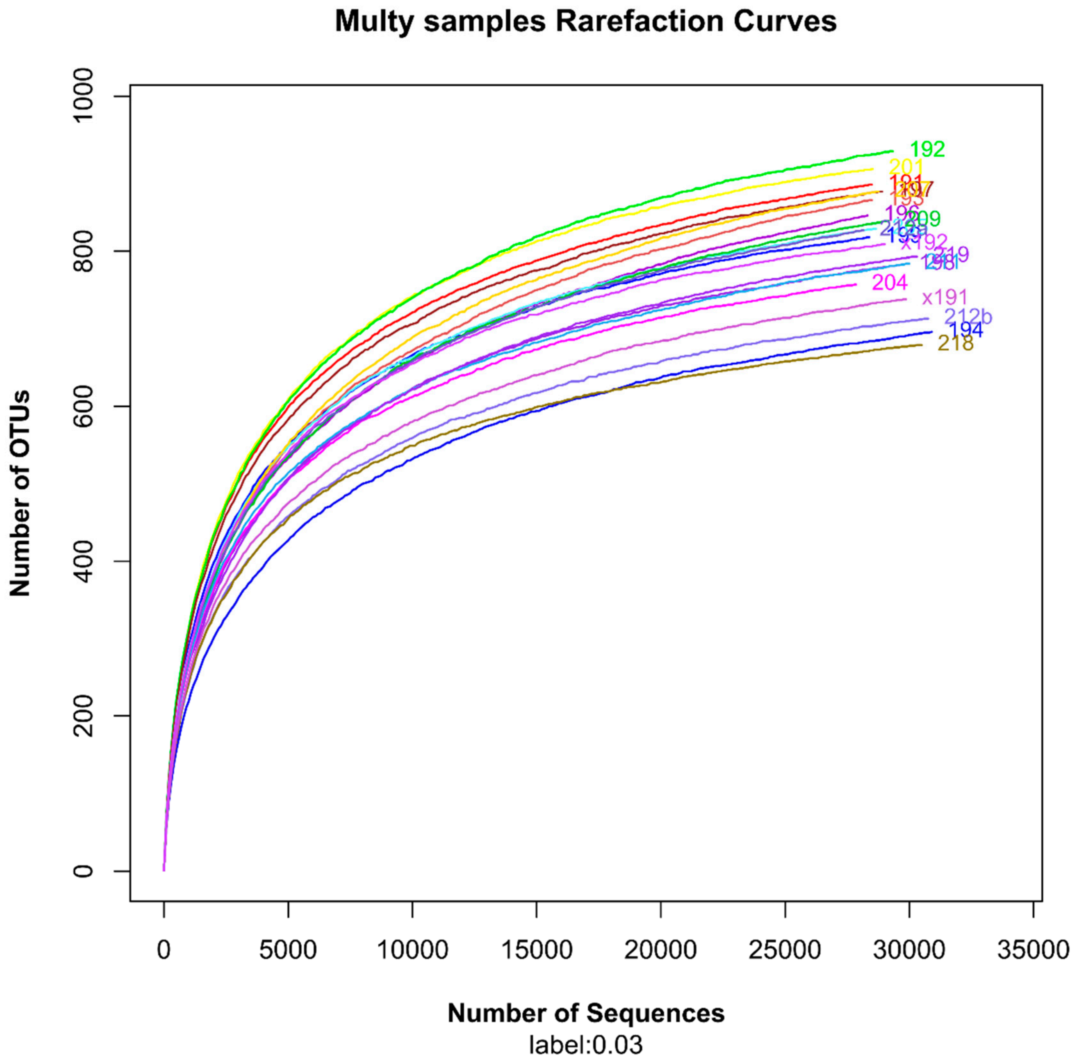

Figure S1. The rarefaction curve of 16S rDNA sequencing

Table S1. Primers used for the quantitative real-time PCR analysis

Target bacterial	Primer sequences [a]	Tm [b] (°C)
Total bacterial (Denman and McSweeney 2006)	F: 5’-CGGCAACGAGCGCAACCC-3’<br>R: 5’-CCATTGTAGCACGTGTGTAGCC-3’	55.3
Ruminococcus albus (Koike and Kobayashi 2001)	F: 5’-CCCTAAAAGCAGTCTTAGTTCG-3’<br>R: 5’-CCTCCTTGCGGTTAGAACA-3’	59.3
Ruminococcus flavefaciens (Koike and Kobayashi 2001)	F: 5’-CGAACGGAGATAATTTGAGTTTACTTAGG-3’<br>R: 5’-CGGTCTCTGTATGTTATGAGGTATTACC-3’	57.7
Fibrobacter succinogenes (Koike and Kobayashi 2001)	F: 5’-GTTCGGAATTACTGGGCGTAAA-3’<br>R: 5’-CGCCTGCCCCTGAACTATC-3’	61.0
Butyrivibrio fibrisolvens (Stevenson and Weimer 2007)	F: 5’-ACCGCATAAGCGCACGGA-3’<br>R: 5’-CGGGTCCATCTTGTACCGATAAAT-3’	55.3
Streptococcus bovis (Tajima et al. 2001)	F: 5’- CTAATACCGCATAACAGCAT -3’<br>R: 5’- AGAAACTTCCTATCTCTAGG -3’	57.0
Prevotella ruminicola (Tajima et al. 2001)	F: 5’-GGTTATCTTGAGTGAGTT-3’<br>R: 5’-CTGATGGCAACTAAAGAA-3’	53.0
Ruminobacter amylophilus (Tajima et al. 2001)	F: 5’- CAACCAGTCGCATTCAGA -3’<br>R: 5’- CACTACTCATGGCAACAT -3’	67.0

[a] F and R represent the forward and reverse primers. [b] Tm represents annealing temperature.

Denman SE, McSweeney CS (2006) Development of a real - time PCR assay for monitoring anaerobic fungal and cellulolytic bacterial populations within the rumen. FEMS Microbiol Ecol 58(3):572-582

Koike S, Kobayashi Y (2001) Development and use of competitive PCR assays for the rumen cellulolytic bacteria: *Fibrobacter succinogenes*, *Ruminococcus albus* and *Ruminococcus flavefaciens*. FEMS Microbiol Lett 204(2):361-366

Stevenson DM, Weimer PJ (2007) Dominance of *Prevotella* and low abundance of classical ruminal bacterial species in the bovine rumen revealed by relative quantification real-time PCR. Appl Microbiol Biotechnol 75(1):165-174

Tajima K, Aminov R, Nagamine T, Matsui H, Nakamura M, Benno Y (2001) Diet-dependent shifts in the bacterial population of the rumen revealed with real-time PCR. Appl Environ Microbiol 67(6):2766-2774

Table S2. Summary of 16S rDNA sequencing data information.

Sample Numbers [a]	Qualified Reads	OUT [b]	Coverage	Chao 1	Ace	Shannon	Simpson
LE194	30900	696	0.996	839.323	795.794	4.715	0.029
LE197	28900	877	0.995	1007.329	970.105	5.423	0.011
LE205	28660	829	0.995	931.374	923.861	5.318	0.011
LE207	28757	877	0.994	1006.767	996.244	5.058	0.020
LE209	29168	839	0.995	981.563	955.904	5.166	0.015
LE219	30308	793	0.996	895.425	881.761	4.806	0.036
ME191	28494	886	0.995	1014.700	985.718	5.443	0.011
ME192	29337	929	0.995	1043.031	1025.195	5.519	0.009
ME193	28498	866	0.994	1020.218	1008.514	5.207	0.014
ME198	29742	783	0.996	889.139	865.570	5.235	0.011
ME199	28388	818	0.995	951.086	911.132	5.302	0.013
ME201	28531	906	0.995	995.202	987.250	5.453	0.010
HE196	28319	846	0.994	1000.800	981.626	5.186	0.014
HE204	27852	757	0.996	839.933	840.705	5.141	0.014
HE218	30500	679	0.996	784.356	757.102	4.705	0.040
HE211	29999	784	0.995	929.043	889.066	5.092	0.018
HE212	28157	827	0.996	830.537	801.231	4.889	0.021
HEx191	29865	738	0.996	855.040	836.634	4.943	0.021

[a] LE: Low dietary energy level group (NEmf = 3.72 MJ/kg, CP = 12.57%). ME: Medium dietary energy level group (NEmf = 4.52 MJ/kg, CP = 12.57%). HE: High dietary energy level group (NEmf = 5.32 MJ/kg, CP = 12.57%). Each group had 6 yaks.

[b] The OTU were identified at a 97% similarity degree.

Table S3. Effects of dietary energy levels on relative abundance of ruminal bacteria (%) at phylum level of yaks.

Items	Groups			SEM	*P*-Value
	LE	ME	HE		
Bacteroidetes	58.33 [a]	57.54 [a]	44.52 [b]	1.834	0.000
Firmicutes	36.22 [b]	36.08 [b]	50.50 [a]	1.932	0.000
Tenericutes	1.46	1.68	1.14	1.221	0.213
Candidate division TM7	0.74	0.66	0.79	0.099	0.894
Lentisphaerae	0.65	0.86	0.74	0.094	0.670
Proteobacteria	0.59	0.88	0.64	0.077	0.268
Synergistetes	0.55	0.35	0.41	0.045	0.155
Others	1.45	1.95	1.27	1.034	0.328

The top 7 abundant phyla were shown (average abundance > 0.5% at least in one group). Each group had 6 yaks. SEM: standard error of the mean. Data with different small letter superscripts within the same row are significantly different ($P < 0.05$).

Table S4. Effects of dietary energy levels on relative abundance of ruminal bacteria (%) at genus level of yaks.

Items	Groups			SEM	*P*-Value
	LE	ME	HE		
Prevotella	9.32	7.08	6.48	1.125	0.582
Succiniclasticum	4.17^{b}	6.99^{ab}	8.66^{a}	0.989	0.047
Saccharofermentans	2.64^{b}	2.27^{b}	4.73^{a}	0.405	0.016
Butyrivibrio	2.03	1.77	2.50	0.196	0.326
Papillibacter	1.21	1.31	0.77	0.124	0.176
Candidatus Saccharimonas	0.70	0.62	1.17	0.164	0.365
Ruminococcus	0.58^{b}	0.82^{ab}	1.15^{a}	0.106	0.040
Pseudobutyrivibrio	0.55^{a}	0.37^{ab}	0.25^{b}	0.044	0.010
Quinella	0.35	0.05	0.09	0.082	0.289
Selenomonas	0.20^{ab}	0.27^{a}	0.09^{b}	0.037	0.039
Victivallis	0.25	0.17	0.34	0.041	0.289
Desulfovibrio	0.22	0.18	0.24	0.019	0.448
Treponema	0.19	0.27	0.18	0.041	0.625
Anaeroplasma	0.19	0.18	0.04	0.038	0.209
Anaerovorax	0.19	0.22	0.24	0.019	0.481
Blautia	0.17^{b}	0.21^{b}	0.77^{a}	0.108	0.029
Bacteroides	0.16	0.15	0.08	0.027	0.484
Oribacterium	0.15	0.17	0.18	0.014	0.685
Fibrobacter	0.09	0.15	0.05	0.021	0.161
Moryella	0.09^{b}	0.09^{b}	0.24^{a}	0.029	0.031
Anaerotruncus	0.08	0.09	0.20	0.023	0.058
Candidatus Hepatincola	0.08	0.08	0.09	0.014	0.963
Faecalibacterium	0.07	0.05	0.14	0.021	0.177
Others	73.43	79.31	71.31	3.452	0.652

The genera populations (average abundance > 0.1% at least in one group) were shown. Each group had 6 yaks. SEM: standard error of the mean. Data with different small letter superscripts within the same row are significantly different ($P < 0.05$)
